# Comparative analysis of perinatal outcomes and birth defects amongst adolescent and older Ugandan mothers: evidence from a hospital-based surveillance database

**DOI:** 10.1186/s12978-021-01115-w

**Published:** 2021-03-04

**Authors:** Robert Serunjogi, Linda Barlow-Mosha, Daniel Mumpe-Mwanja, Dhelia Williamson, Diana Valencia, Sarah C. Tinker, Michelle R. Adler, Joyce Namale-Matovu, Dennis Kalibbala, Jolly Nankunda, Evelyn Nabunya, Doreen Birabwa-Male, Josaphat Byamugisha, Philippa Musoke

**Affiliations:** 1 Makerere University-Johns Hopkins University Research Collaboration, P. O. Box 23491, Kampala, Uganda; 2 US Centers for Disease Control and Prevention (CDC), Atlanta, GA USA; 3 US Centers for Disease Control and Prevention (CDC), Kampala, Uganda; 4 Department of Paediatrics and Child Health, College of Health Sciences, Makerere University, Kampala, Uganda; 5 Department of Obstetrics and Gynaecology, College of Health Sciences, Makerere University Kampala, Kampala, Uganda

**Keywords:** Adolescent, Birth outcomes, Birth defects, Gastroschisis, Low birth weight, Early neonatal death, Preterm, Hospital-based surveillance, Sub-Saharan Africa, Uganda

## Abstract

**Background:**

Uganda has one of the highest adolescent pregnancy rates in sub-Saharan Africa. We compared the risk of adverse birth outcomes between adolescents (age 12–19 years) and mothers (age 20–34 years) in four urban hospitals.

**Methods:**

Maternal demographics, HIV status, and birth outcomes of all live births, stillbirths, and spontaneous abortions delivered from August 2015 to December 2018 were extracted from a hospital-based birth defects surveillance database. Differences in the distributions of maternal and infant characteristics by maternal age groups were tested with Pearson’s chi-square. Adjusted odds ratios (aORs) and 95% confidence intervals (CI) were calculated using logistic regression to compare the prevalence of adverse birth outcomes among adolescents to mothers 20–34 years.

**Results:**

A total of 100,189 births were analyzed, with 11.1% among adolescent mothers and 89.0% among older mothers. Adolescent mothers had an increased risk of preterm delivery (aOR: 1.14; CI 1.06–1.23), low birth weight (aOR: 1.46; CI 1.34–1.59), and early neonatal deaths (aOR: 1.58; CI 1.23–2.02). Newborns of adolescent mothers had an increased risk of major external birth defects (aOR: 1.33; CI 1.02–1.76), specifically, gastroschisis (aOR: 3.20; CI 1.12–9.13) compared to mothers 20–34 years. The difference between the prevalence of gastroschisis among adolescent mothers (7.3 per 10,000 births; 95% CI 3.7–14.3) was statistically significant when compared to mothers 20–34 years (1.6 per 10,000 births; 95% CI 0.9–2.6).

**Conclusions:**

This study found that adolescent mothers had an increased risk for several adverse birth outcomes compared to mothers 20–34 years, similar to findings in the region and globally. Interventions are needed to improve birth outcomes in this vulnerable population.

## Plain English Summary

Adolescent pregnancies are a global problem occurring in high-, middle-, and low-income countries with Uganda having one of the highest adolescent pregnancy rates in sub-Saharan Africa. We compared the risk of adverse birth outcomes, including major external birth defects, between adolescents, (age 12–19 years) and mothers (age 20–34 years) in four urban hospitals.

All informative births, including live births, stillbirths, and spontaneous abortions; regardless of gestational age, delivered at four selected hospitals in Kampala from August 2015 to December 2018 were examined. Demographic data were obtained by midwives through maternal interviews and review of hospital patient notes.

Of the 100,189 births, 11.0% were among adolescent mothers and 89.0% among mothers (20–34 years). Adolescent mothers were more likely than mothers (20–34 years) to have an infant with preterm delivery, low birth weight, early neonatal death, and major external birth defects. Adolescent pregnancies were also associated with an increased risk of gastroschisis when compared to mothers (20–34 years).

In conclusion, this study found that adolescent mothers had an increased risk for several adverse birth outcomes compared to mothers 20–34 years. Research on the potential underlying causes or mechanisms for these adverse outcomes among adolescent births is necessary to identify possible interventions.

## Introduction

Pregnancies among 15–19 year-old females account for 16 million (11%) births worldwide yet they contribute to 23% of the maternal disease burden attributed to pregnancy and childbirth [1, 2]. The highest prevalence of adolescent pregnancy is found in the sub-Saharan African region, with birth rates of 101 births per 1000 females aged 15–19 years in 2018, higher than the global adolescent birth rate of 44 per 1000 [3].

Uganda has one of the youngest populations in sub-Saharan Africa, with children and adolescents 12–19 years constituting more than half (55%) of the population in 2014 [4], and one of the highest adolescent pregnancy rates (25%) in sub-Saharan Africa [5]. Despite a decline in the fertility rate in Uganda from 6.9 in 2000 to 5.4 in 2016, and an increase in the use of modern contraception from 18% in 2000 to 35% in 2016, adolescent pregnancy remains a challenge with only 7.6% of adolescents having access to contraceptives [5]. Adolescents have also been reported to be less likely to prepare for birth and even be less knowledgeable about obstetric danger signs compared to older mothers who were not knowledgeable [6], potentially increasing the risk of adverse birth outcomes.

Although several studies have found a higher risk of adverse birth outcomes such as preterm birth, low birth weight (LBW), and early neonatal deaths (ENND), with adolescent births, [1, 7–13], some studies have not found an association for some adverse birth outcomes [14–19]. Some possible reasons for such differences in results could be the sample size or categorization of age groups among the adolescents and comparative age group. In addition, a systematic literature review and meta-analysis on adolescent childbearing in Sub-Saharan Africa by Gronvik et al. (2018) [19] showed that most studies were primarily hospital or health clinic-based patient record reviews and therefore may not be representative of the general population.

Most studies [7, 10, 14, 19] that have reported birth outcomes among adolescent births in Sub-Saharan Africa have not reported the burden of major external birth defects among infants born to adolescents. There have also been a limited number of studies [21, 22] in the Sub-Saharan Africa region that have documented the prevalence and the risks of major external birth defects among adolescent births in comparison with births from mothers over 19 years of age. This may be as a result of limited data on these conditions probably as a result of inadequate birth defect registry systems [20].

Therefore, using a large dataset obtained from an ongoing hospital-based birth defect surveillance study, we compared the occurrence of adverse birth outcomes (preterm birth, LBW, and ENND), including the rates and prevalence of specific major external birth defects among adolescent mothers (12–19 years) and mothers (20–34 years) in Uganda, a low-middle income setting. The findings from this study would therefore be used as a benchmark for researchers and policymakers to understand the current estimate of the burden of adverse birth outcomes among adolescent births in a low-income Sub-Saharan African country.

## Methods

We extracted and analyzed verified data collected between August 2015 and December 2018 from an ongoing birth defects surveillance system implemented at four major hospitals in Kampala, Uganda [23]. These hospitals have approximately 50,000 births annually, which make up more than 55% of all births in Kampala. The details of the birth defects surveillance system are described elsewhere [23]. Briefly, this birth defects surveillance system collected information from hospital records including: demographic (maternal age, delivery site), maternal health (maternal HIV status, obstetric history), and birth outcome (mode of delivery, pregnancy outcome, infant sex, gestational age, and infant examinations). Information on maternal HIV status and antiretroviral therapy was obtained from antenatal records and inpatient hospital records. Information on all live births, stillbirths, and spontaneous abortions was collected between the time of birth and discharge which usually occurs within the first 24 h after delivery [23]. Infants born outside the four hospitals and uninformative macerated stillbirths were not included in the surveillance system.

We defined adolescent births for this analysis as those occurring in women 12–19 years of age at delivery and the comparative group as births among women 20–34 years of age at delivery. There were no births to women younger than 12 years of age, and births of mothers ≥ 35 years of age were excluded because the risk for adverse obstetrical and perinatal outcomes has been shown to increase over age 34 [24, 25].

We defined gestational age as the interval between the date of delivery and the last menstrual period (LMP) in completed weeks; if the LMP was unknown or missing, a clinical estimate of gestational age was used, such as estimates from fundal height or abdominal ultrasound. We defined preterm delivery as live births occurring at gestations of less than 37 weeks. Low birth weight (LBW) was defined as an infant weighing less than 2,500 g measured within 24 h after birth using digital scales among term (≥ 37 weeks) live births. Early neonatal death (ENND) was defined as death among live neonates born at term during the first 48 h or before the mother was discharged from the hospital. Stillbirth was defined as a baby born with no signs of life at or after 28 weeks’ gestation, while a spontaneous abortion was defined as fetal death at less than 28 weeks' gestation. Birth defects were confirmed through bedside examination by a physician and review of photographs, narrative descriptions, and or drawings by a birth defects expert who verified or reassigned the diagnosis code. Details of the birth defect ascertainment and classification have been described previously [23]. The definition of birth defects included in this analysis can be found in the Additional file 1.

Data were analyzed using STATA version 15 statistical software (StataCorp. 2017. College Station, TX: StataCorp LLC). Descriptive statistics of maternal and infant characteristics by maternal age group were calculated as frequencies and percentages, and the differences between proportions were tested with Pearson’s chi-square test.

We used multivariable logistic regression analysis to estimate crude and adjusted odds ratios (cORs and aORs, respectively) along with their 95% confidence intervals (CIs) for the associations between adolescent births, and adverse birth outcomes with the 20–34 years age group as the reference. Separate multivariable logistic regression models were generated for preterm birth, LBW, ENND, each major birth defect category (neural tube defects, malformations of the eyes and ears, orofacial clefts, and malformations of the musculoskeletal system), and each of the 16 specific birth defects. The analysis of preterm birth was limited to live births; while that of LBW and ENND was limited to term live births. The following covariates were considered for adjustment: parity, mode of delivery, singleton/multiple delivery, number of antenatal visits, and initiation time of prenatal care. The specific covariates used in each model were selected based on previous studies [7, 14, 26–28], and excluded possible collider variables usingdirected acyclic graphs (DAGs) to evaluate confounding [29]. The variables considered in the models are shown in Table 1 and the final variables included in the models are listed as a footnote in Table 2.

**Table 1:** Maternal and reproductive characteristics of adolescent mothers 12–19 and mothers 20–34 years of age

	Total, n (%)	Maternal age, n (%)		p-Value
		12–19 years	20–34 years	
No. of births	100,189 (100)	11,028 (11.0)	89,161 (89.0)	–
No. of mothers	**96,938 (100)**	**10,783 (11.1)**	**86,155 (88.9)**	–
Maternal age				
Median; Inter-quartile range (IQR)	25; 22–29	18; 18–19	26; 23–29	–
Hospital^b^				
Lubaga	6410 (6.4)	134 (1.2)	6276 (7.0)	< 0.001
Mengo	7905 (7.9)	111 (1.0)	7794 (8.7)	
Nsambya	7531 (7.5)	99 (0.9)	7432 (8.3)	
Mulago national referral	78,343 (78.2)	10,684 (96.9)	67,659 (75.9)	
Maternal HIV status^a^				
Positive	8167 (8.4)	480 (4.5)	7687 (8.9)	< 0.001
Negative	88,631 (91.4)	10,282 (95.3)	78,349 (91.0)	
Unknown	140 (0.1)	21 (0.2)	119 (0.1)	
Maternal antiretroviral therapy (ART) at delivery^β^				
Yes	7786 (95.3)	438 (91.3)	7348 (95.6)	< 0.001
No	381 (4.7)	42 (8.8)	339 (4.4)	
Maternal timing of initiation on ART^∏^				
Before conception	4161 (53.4)	133 (30.4)	4028 (54.8)	< 0.001
After conception	3625 (46.6)	305 (69.6)	3320 (45.2)	
Mother referred from other health center^a^				
Yes	44,700 (46.1)	7541 (69.9)	37,159 (43.1)	< 0.001
No	52,238 (53.9)	3242 (30.1)	48,996 (56.9)	
Maternal parity^a^				
Primipara (1)	32,765 (33.8)	9023 (83.7)	23,742 (27.6)	< 0.001
Multipara (≥ 2)	64,173 (66.2)	1760 (16.3)	62,413 (72.4)	
Mode of delivery^b^				
Vaginal	68,756 (68.6)	8575 (77.8)	60,181 (67.5)	< 0.001
Caesarean section	31,433 (31.4)	2453 (22.2)	28,980 (32.5)	
Singleton/multiple deliveries^b^				
Singleton	93,548 (93.4)	10,516 (95.4)	83,032 (93.1)	< 0.001
Multiple	6641 (6.6)	512 (4.6)	6129 (6.9)	
Received antenatal care (maternal)^a^				
Yes	94,734 (97.7)	10,403 (96.5)	84,331 (97.9)	< 0.001
No	2204 (2.3)	380 (3.5)	1824 (2.1)	
Timing of first antenatal care (ANC) visit^a^^, ξ^				
ANC within 1st Trimester	6446 (7.9)	580 (6.6)	5866 (8.0)	< 0.001
ANC within 2nd Trimester	36,783 (44.9)	3976 (45.3)	32,807 (44.8)	
ANC within 3rd Trimester	38,696 (47.2)	4217 (48.1)	34,479 (47.1)	
Number of maternal antenatal visits^a^^,π^				
No ANC Visit	2204 (2.3)	380 (3.5)	1824 (2.1)	< 0.001
1–3 Visits	52,764 (54.6)	6626 (61.5)	46,138 (53.7)	
4 + Visits	41,731 (43.2)	3761 (34.9)	37,970 (44.2)	

^a^Denominator is the number of mothers

^b^Denominator is the number of births

^β^Denominator is the number of HIV positive mothers (n = 8167)

^**∏**^Denominator is the number of HIV positive mothers on ART (n = 7786)

^*ξ*^10,605 Mothers missing date of first ANC visit

^*π*^239 Mothers missing the number of ANC visits

**Table 2:** Comparison of perinatal outcomes between adolescent mothers 12–19 and mothers 20–34 years of age

	Total, n (%)	Maternal age, n (%)		cOR (95% CI)*	p-Value^c^	aOR (95% CI) *	p-value^d^
		12–19 Years	20–34 Years				
*Gestational age*^*a*^							
< 37 weeks	8564 (9.0)	1068 (10.2)	7496 (8.8)	1.18 (1.10–1.26)	< 0.001	1.14 (1.06–1.23)	0.001
$\ge$ 37 weeks	86,839 (91.0)	9358 (89.8)	77,481 (91.2)	1		1	
*Birth outcome*							
Live birth	95,403 (95.2)	10,426 (94.5)	84,977 (95.3)	1		1	
Stillbirth	3102 (3.1)	359 (3.3)	2743 (3.1)	1.07 (0.95–1.19)	0.258	1.08 (0.95–1.22)	0.230
Spontaneous Abortion	1684 (1.7)	243 (2.2)	1441 (1.6)	1.37 (1.19–1.58)	< 0.001	0.94 (0.83–1.11)	0.488
*Infant birth weight (*$\ge$* 37 weeks)*^*a*^							
< 2500 g	6572 (7.6)	986 (10.5)	5586 (7.2)	1.51 (1.41–1.63)	< 0.001	1.46 (1.34–1.59)	< 0.001
$\ge$ 2500 g	80,267 (92.4)	8372 (89.5)	71,895 (92.8)	1		1	
*ENND (*$\ge$* 37 weeks)*^*a,b*^							
Yes	441 (0.5)	82 (1.0)	359 (0.5)	1.96 (1.57–2.45)	< 0.001	1.58 (1.23–2.02)	< 0.001
No	82,159 (99.5)	8511 (99.0)	73,648 (99.5)	1		1	
*Birth defect*							
No	99,674 (99.5)	10,954 (99.3)	88,720 (99.5)	1		1	
Yes^¥^	515 (0.5)	74 (0.7)	441 (0.5)	1.36 (1.06–1.74)	0.015	1.36 (1.02–1.80)	0.032

^a^Live births only (n= 95,403)

^b^Early neonatal death (ENND); term births (n= 86,839)

^c^p-value for cOR

^d^p-value for aOR

*The cOR (95% CI) and aOR (95% CI) were calculated with 20-34 years as the reference age group

Gestational age model was restricted to live births only with adjustment for parity, mode of delivery, singleton/multiple deliveries, and number of antenatal visits

Birth outcome model was adjusted for parity, mode of delivery and number of antenatal visits

Early neonatal death model was restricted to full-term infants (gestation ≥37 weeks) and adjusted for parity, mode of delivery and number of antenatal visits

Birth weight model was restricted to full-term infants (gestation ≥37 weeks) and adjusted for parity, mode of delivery, singleton/multiple deliveries and number of antenatal visits

Overall birth defect model was adjusted for parity, mode of delivery, singleton/multiple births and number of antenatal visits. ^¥^Newborns with at least one of the sixteen major external birth defects of interest to the study

Birth prevalence per 10,000 births for seven categories of major external birth defects and 16 specific birth defects [23] was calculated by each maternal age group along with 95% Wilson’s CIs.

Those defects that were considered genetic, for example, anencephaly with Spina Bifida or OEIS complex (omphalocele-exstrophy-imperforate anus-spinal defects) which comprises a combination of defects including omphalocele, exstrophy of the cloaca, imperforate anus, and spinal defects, were included in prevalence estimates, but not be included in etiologic or risk factor analysis since the etiology of the defects are known or suspected [30, 31].

## Results

**Fig. 1:**
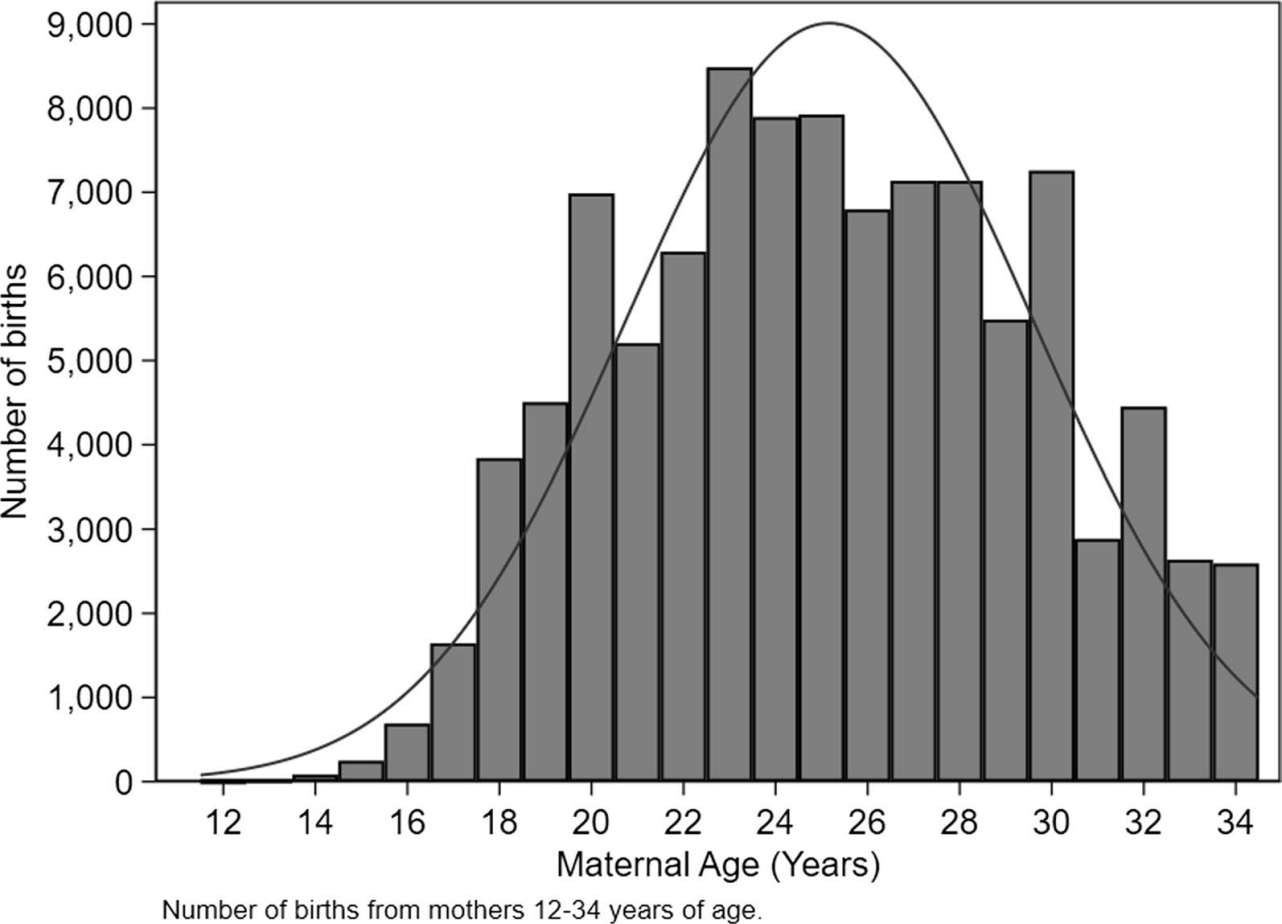
Distribution of births by maternal age among mothers 12–34 years of age

A total of 96,938 pregnancies with 100,189 births among mothers 12 to 34 years of age were captured. Of these, 11,028 (11.0%) births were among adolescent mothers and 89,161 (89.0%) births were among mothers (20–34 years). The age distribution of the study population of all mothers aged 12 to 34 years is shown in Fig. 1.

Table 1 shows the maternal and infant characteristics by age group. The proportion of mothers with HIV infection was significantly lower in adolescent mothers (p < 0.001) but a significantly higher proportion of HIV-infected adolescents had not been initiated on antiretroviral therapy (ART) by the time of delivery compared to mothers (20–34 years) (p < 0.001). Adolescent mothers were less likely to have attended any antenatal care (ANC), attended the recommended four or more antenatal visits [32], or attended the first antenatal visit within the first trimester (p < 0.001) compared to mothers (20–34 years). Also, adolescents were more likely to have been referred from another health center for delivery, contributing 70% of referred women. Adolescent mothers were also more likely than mothers (20–34 years) to be primipara, have vaginal deliveries, and have singleton deliveries (p < 0.001).

Adolescent mothers were significantly more likely than mothers (20–34 years) to have preterm (< 37 weeks) live births (aOR: 1.14; 95% CI 1.06–1.23, p = 0.001) (Table 2). Among live births delivered at term, adolescents were at higher risk of delivering a LBW infant (aOR: 1.46; 95% CI 1.34–1.59; p < 0.001) and early neonatal death (aOR: 1.58; 95% CI 1.23–2.02; p < 0.001) (Table 2). Adolescents were also more likely to have a spontaneous abortion (cOR:1.37 95% CI 1.19–1.58; p < 0.001), but after adjusting for confounders the association was not statistically significant (Table 2).

**Fig. 2:**
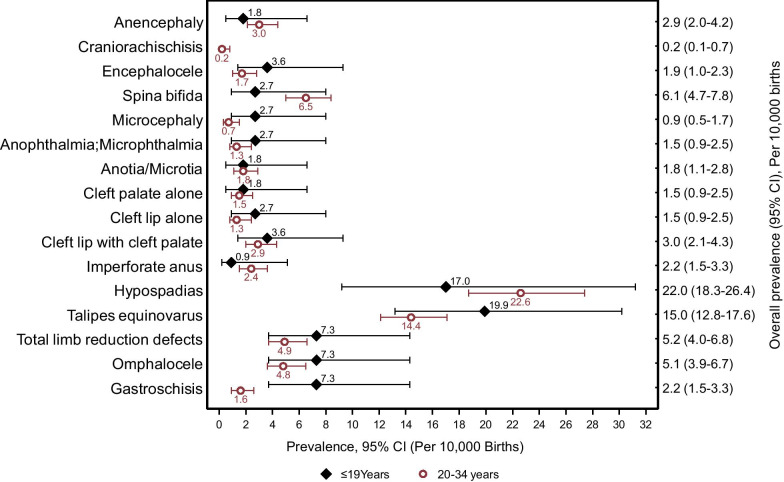
Birth Prevalence per 10,000 births, 95% CI of major external birth defects by maternal age groups, Kampala, Uganda

Adolescent mothers had a higher prevalence of birth defects (67.1 per 10,000 births, 95% CI 53.5–84.2) compared to mothers (20–34 years) (49.7 per 10,000 births, 95% CI 45.3–54.5). The odds of major external birth defects were higher among adolescents in comparison to mothers (20–34 years) (aOR: 1.36; 95% CI 1.02–1.80; p = 0.032). Talipes equinovarus was the most prevalent major external birth defect among adolescent mothers (19.9 per 10,000 births; 95% CI 13.2–30.2) (Fig. 1) The prevalence estimates (per 10,000 births) of 10 birth defects (Encephalocele, microcephaly, anophthalmia; microphthalmia, all oral-facial clefts, talipes equinovarus, limb reduction defects, omphalocele, and gastroschisis) were higher among adolescent mothers, however, only the difference between the prevalence of gastroschisis among adolescent mothers (7.3 per 10,000 births; 95% CI 3.7–14.3) was statistically significant when compared to mothers (20–34 years) (1.6 per 10,000 births; 95% CI 0.9–2.6) (Fig. 2).

Adolescent mothers were significantly more likely to have an infant born with microcephaly and gastroschisis. However, after adjustment for parity and initiation time of prenatal care, only gastroschisis (aOR: 3.20; 95% CI 1.12–9.13) remained significantly associated with adolescent pregnancy (Table 3). Musculoskeletal defects (aOR: 1.69; 95% CI 1.15–2.50) and malformations of eyes and ears (aOR: 3.09; 95% CI 1.01–9.42) were also significantly higher among adolescent births compared to those from mothers (20–34 years) (Table 3).

**Table 3:** Birth defects among adolescent mothers 12–19 and mothers 20–34 years of age

ICD-10 RCPCH code^a^	Birth defects	Number of defects		cOR (95% CI)	aOR (95% CI)^d^	p-value
		12–19 years	20–34 years			
Neural tube defects (NTD)*		9	95	0.77 (0.39–1.52)	0.63 (0.27–1.52)	0.311
Q00.0	Anencephaly	2	27	0.60 (0.14–2.52)	0.64 (0.14–2.90)	0.559
Q00.1	Craniorachischisis	0	2	na	na	na
Q01.0–Q01.2, Q01.8–Q01.9	Encephalocele	4	11	2.94 (0.94–9.24)	1.43 (0.27–7.43)	0.673
Q05.0–Q05.9	Spina bifida	3	56	0.43 (0.14–1.38)	0.40 (0.08–1.67)	0.202
Q02	Microcephaly	3	6	4.04 (1.01–16.17) ^β^	4.54 (0.81–25.39)	0.085
Malformations of eyes and ears		5	28	1.44 (0.56–3.74)	3.09 (1.01–9.42)	0.047
Q11–Q11.1; Q11.2	Anophthalmia; Microphthalmia	3	12	2.02 (0.57–7.16)	3.21 (0.71–14.38)	0.128
Q16.0; Q17.2	Anotia; Microtia	2	16	1.01 (0.23–4.40)	2.94 (0.55–15.72)	0.206
Orofacial clefts^b^		9	51	1.43 (0.70–2.90)	1.28 (0.57–2.91)	0.549
Q35.1–Q35.9, Q38.5, Q87.0	Cleft palate	2	13	1.24 (0.28–5.51)	0.71 (0.08–6.12)	0.752
Q36.0, Q36.9	Cleft lip alone	3	12	2.02 (0.57–7.16)	2.54 (0.59–11.50)	0.213
Q37.0–Q37.9	Cleft lip + palate	4	26	1.24 (0.43–3.56)	1.09 (0.35–3.41)	0.877
Q42.3	Imperforate anus	1	20	0.40 (0.05–3.01)	1.06 (0.12–9.08)	0.960
Q54.0–Q54.3, Q54.8–Q54.9	Hypospadias^c^	10	104	0.75 (0.39–1.44)	0.63 (0.29–1.34)	0.230
Musculoskeletal system*		45	214	1.70 (1.23–2.35)^β^	1.69 (1.15–2.50)	0.008
Q66.0, Q66.8	Talipes equinovarus	22	128	1.41 (0.89–2.22)	1.33 (0.77–2.30)	0.309
Q71.0–Q73.8	Total limb reduction	8	44	1.47 (0.69–3.12)	1.75 (0.67–4.56)	0.249
Q79.2	Omphalocele	8	41	1.58 (0.74–3.37)	2.17 (0.92–5.18)	0.078
Q79.3	Gastroschisis	8	14	4.62 (1.93–11.02)^β^	3.20 (1.12–9.13)	0.030

* Some infants had more than one type of defect in the neural tube defects and musculoskeletal system categories

^a^International Classification of Disease 10, Royal College of Paediatrics and Child Health (ICD-10 RCPCH), used to specifically clasify the types of defects included  [49]

^b^Excluded Q36.1 (medial Cleft lip) because it is suggestive of underlying brain malformation (holoprosencephaly) and chromosomal syndrome [50]

^β^Statistically significant at p<0.05

na - Prevalence, cOR, aOR, and 95% confidence intervals that cannot be calculated

^c^Denominator for males: N=51,922; 12-19 Years (n= 5,896); 20-34 Years (n= 46,026)

^d^Covariates for the birth defect models: parity and initiation time of prenatal care

## Discussion

In this study, we observed that adolescent mothers were more likely to have an infant with the adverse birth outcome of preterm delivery, LBW, ENND, or a major external birth defect such as gastroschisis as compared to mothers 20–34 years. Previous studies have also found an increased risk for preterm delivery in adolescent births [7, 14, 19], which could be attributable to the maternal–fetal competition for nutrients that arises when pregnancy coincides with continuing or incomplete growth in adolescents. Our study finds that adolescent mothers were more likely to deliver LBW babies, and is consistent with results from the Uganda Demographic Health Survey (UDHS) 2011 [33], and several other studies in sub-Saharan Africa [14, 34–36]. That UDHS also identified infants born with LBW to be at increased risk of neonatal death [37], highlighting the risks associated with LBW in this population. The LBW observed among infants born to adolescent mothers could have been due to factors such as inadequate maternal nutrition, or the related but distinct issue of inadequate weight gain during pregnancy [26], which were not assessed in our study.

Comparable to findings from a study exploring the impact of early motherhood on neonatal mortality in 45 low and middle-income countries [8], our study showed that ENNDs in full-term babies occurred more frequently among adolescent mothers. In contrast, a World Health Organization (WHO) multi-country survey across 29 countries in Africa, Asia, Latin America, and the Middle East found that ENND among infants born to adolescent mothers was not significantly different from mothers aged 20–24 years, after adjustment for gestational age and birth weight [7]. This difference may be related to restriction in the WHO study to mothers aged 24 years or younger who gave birth to an infant of at least 22 weeks’ gestation as compared to mothers ≤ 34 years in our analysis, and the WHO study’s classification of ENND as intra-hospital deaths that occurred within 7 days after birth as compared to deaths within 48 h in our analysis.

In this study, adolescent mothers were more likely to deliver a newborn with a birth defect when compared with mothers 20–34 years. These findings are consistent with findings from studies in North America and Europe [38, 39]. Our finding of a higher birth defects prevalence estimate (per 10,000 births) among adolescent mothers compared to older mothers is consistent with findings from a population-based prevalence study using data from EUROCAT congenital anomaly registers in 23 regions of Europe in 15 countries [39]. However, Zile and Villerusa et al. (2013), from a study based on data from the Medical Birth Register in Latvia differed showing that the prevalence of birth defects was instead higher for mothers aged 20–34 years as compared to adolescent mothers [40]. The difference could however be attributed to the fact that our study’s prevalence estimates included births from all live births, stillbirths, and spontaneous abortions while Zile and Villerusa et al. (2013) included only live births and also included other defects/syndromes and chromosomal defects.

Although the number for some birth defects were small in our study, our findings suggest that gastroschisis was significantly higher among adolescent mothers when compared to mothers 20–34 years. The strong association between adolescents births with gastroschisis has also been reported by other studies [28, 38, 39, 41]. While comparing gastroschisis to other congenital anomalies, Given et al. (2017) reported sexually transmitted infections, and continuation of oral contraceptives in early pregnancy, as preventable risk factors [42]. However, we were not able to assess these factors in this study.

Our study also found that adolescent mothers were associated with increased odds of musculoskeletal defects as well as malformations of eyes and ears combined. In one retrospective cohort study in the United States of America, [38] Chen et al. (2007) found increased odds of musculoskeletal defects, however, the study included some other defects within the category, specifically, polydactyly/syndactyly/adactyly, diaphragmatic hernia, integumentary anomalies.

We also found that a significantly higher proportion of HIV-infected adolescents were not on ART at conception or delivery compared to women 20–34 years, which is consistent with findings from the Uganda Population-Based HIV Impact Household-based National Survey [43]. Maternal HIV infection has been shown to be associated with increased rates of adverse pregnancy outcomes such as LBW, prematurity, and ENND [44], and the lower prevalence of ART use among HIV-infected adolescents would further exacerbate the situation because it translates to a potential increased risk of MTCT of HIV among adolescents compared to mothers (20–34 years) justifying the need to strengthen services for this population [45].

## Study strengths and limitations

This study’s strengths include a large sample size, which made it possible to assess the association between adolescent births and possible risk factors of adverse birth outcomes. In addition, our study used an active birth defects case ascertainment and collection of data to ensure accuracy and improved birth defect detection and reporting versus extraction of data from medical records. Also, the physical examination of newborns by trained staff and several levels of external birth defect review ensured consistent birth defect classification and coding.

Unlike other studies that only include live births [38, 40], this study included stillbirths, spontaneous abortions, and live births which minimized selection bias especially since some structural birth defects commonly occur among stillbirths thereby giving more accurate risks and birth prevalence estimates among the different age groups.

Study limitations include surveillance activities being conducted at four major urban hospitals located in the capital city and is not representative of adolescent births nationally [5]. However, since 55% of the births in Kampala were at these four hospitals, and one of them (Mulago National Referral Hospital) contributed 60.0% of the total births [23], they provide a fair representation of births nationally. Secondly, because infants were not followed post-discharge, we captured only ENND that occurred within 48 h of birth. The standard definition of ENND is death within seven days of delivery so infants that died between discharge and seven days of life was not accounted for, resulting in a possible underestimation of ENND.

In addition, this study did not control for several risk factors known to influence reproductive health outcomes such as social-economic status, level of education, tobacco smoking, alcohol drinking, maternal nutrition, and the use of folic acid since this information was not captured in the surveillance [28, 46].

Finally, it has been demonstrated that adolescents are not a homogeneous group, and therefore differ in their emotional or cognitive development [47], and that categorizing adolescents into one age group could withhold full knowledge of the most vulnerable age groups associated with adverse birth outcomes. However, we lumped the adolescent age-group into one group of mothers less than 19 years of age because our study had small segregated sample sizes within the finer age group categories, especially in the 12–14-year-old group. Therefore, further research from this ongoing surveillance will seek to investigate the risk factors associated with the different adolescent age groups.

## Conclusion

Our study is one of the few studies reporting adverse birth outcomes among adolescents in Uganda Our results corroborate previous findings in both developed and developing countries on birth outcomes and demonstrate that adolescent births are at risk for several neonatal adverse birth outcomes. With the growing population and high rates of adolescent births in Africa, the number of adverse birth outcomes is likely to increase and thereby remains a key public health concern [5].

Further research on individual, socio-cultural, environmental, economic, and health service-related factors are required to identify practicable and scalable measures to decrease adolescent pregnancy and to identify and reduce obstacles that discourage the use of qualified antenatal services, that would prevent or reduce adverse reproductive outcomes such as neonatal deaths, low birth weight, birth defects, and mother to child transmission of HIV. The establishment of dedicated adolescent-friendly antenatal care programs would help improve neonatal and adolescent health [48], and, better understand associated risk factors and the impact of younger maternal age on pregnancy outcomes. It is critical to monitor trends in birth outcomes and prevalence of major external birth defects across age groups to inform health-care policies and to plan for needed services among the affected population. Research on the potential underlying causes or mechanisms for these adverse outcomes among adolescent births is necessary to identify possible interventions.

## Supplementary Information

**Additional file 1.** Definition of Birth defects

## Data Availability

Not applicable.

## Abbreviations

cOR: Crudes odds ratio
aORs: Adjusted odds ratios
CI: Confidence intervals
LBW: Low birth weight
ENND: Early neonatal deaths
MTCT: Mother to child transmission
ANC: Antenatal care
ART: Antiretroviral therapy
WHO: World Health Organization
CDC: Centers for Diseases Control and Prevention
HIV: Human immunodeficiency virus
LMP: Last menstrual period
US: United States
IQR: Inter-quartile range
NTD: Neural tube defects
ICD-10 RCPCH: International Classification of Disease 10, Royal College of Paediatrics and Child Health
OEIS: Omphalocele-exstrophy-imperforate anus-spinal defects
