# Supplementary material for: Comparative analysis of perinatal outcomes and birth defects amongst adolescent and older Ugandan mothers: evidence from a hospital-based surveillance database
Source: Reprod Health. 2021 Mar 4;18:56. doi: 10.1186/s12978-021-01115-w (PMC7934544; doi:10.1186/s12978-021-01115-w)
Supplement: Supplementary file 1 — Additional file 1. Definition of Birth defects [file 12978_2021_1115_MOESM1_ESM.docx]

Definition of Birth defects

| **Birth Defect (ICD-10 RCPCH^a^)** | **Definition/Description** |
| --- | --- |
| **Anencephaly (Q00.0)** | A congenital malformation characterized by the total or partial absence of the cranium vault, the covering skin, and the brain missing or reduced to small mass. Includes craniorachischisis and infants with iniencephaly and other neural tube defects such as encephalocele and open spina bifida, when associated with anencephaly. Excludes acephaly, that is, the absence of head observed in amorphous acardiac twins. |
| **Craniorachischisis (Q00.1)** | Craniorachischisis refers to the presence of anencephaly with a contiguous spine defect without meninges covering the neural tissue (rachischisis). It might be limited to the cervical region or affect the entire spine. Newborns with craniorachischisis also might have spinal retroflection resembling the body habitus of newborns with iniencephaly. |
| **Encephalocele (Q01.0-Q01.2, Q01.8-Q01.9)** | A congenital malformation characterized by herniation of the brain and or meninges through a defect in the skull. Encephalocele is not counted when present with spina bifida. |
| **Microcephaly (Q02)** | A congenitally small cranium, defined by an occipitofrontal circumference (OFC), 3 standard deviations below the age‑ and sex‑appropriate distribution curves. Excludes microcephaly associated with anencephaly or encephalocele. |
| **Spina bifida (Q05.0-Q05.9)** | A family of congenital malformation defects in the closure of the spinal column characterized by herniation or exposure of the spinal cord and or meninges through an incompletely closed spine. Includes: meningocele, meningomyelocele, myelocele, myelomeningocele, and rachischisis. Spina bifida is not counted when present with anencephaly. Excludes spina bifida occulta, and sacrococcygeal teratoma without dysraphism. |
| **Anophthalmia (Q11-Q11.1) Microphthalmia (Q11.2)** | Absent or small eyes. Some normal adnexal elements and eyelids are usually present. In microphthalmia, the corneal diameter is usually less than 10mm, and the anteroposterior diameter of the globe is less than 20 mm. |
| **Anotia/Microtia (Q16.0, Q17.2)** | A congenital malformation characterized by absent parts of the pinna (with or without atresia of the ear canal) commonly expressed in grades (I-IV) of which the extreme form (grade IV) is Anotia, absence of pinna. Excludes small, normally shaped ears, imperforate auditory meatus with a normal pinna, dysplastic, and low set ears. |
| **Cleft palate alone (Q35.1-Q35.9, Q38.5, Q87.0)** | A congenital malformation characterized by a closure defect of the hard and or soft palate behind the foramen incisivum without cleft lip. Includes, submucous cleft palate. Excludes cleft palate with cleft lip, cleft uvula, functional short palate, and high narrow palate. |
| **Cleft lip with or without cleft palate (Q36.0, Q36.9) (Q37.0-Q37.9)** | A congenital malformation characterized by partial or complete clefting of the upper lip, with or without clefting of the alveolar ridge or the hard palate. Excludes midline cleft or upper or lower lip and oblique facial fissure (going towards the eye). |
| **Imperforate anus (Q42.3)** | A congenital malformation characterized by the absence of continuity of the anorectal canal or of communication between rectum and anus, or narrowing of the anal canal, with or without fistula to neighboring organs. The rectum may terminate as a blind sac, with no communication to the colon, or may open to the urethra, bladder, or vagina. Excludes mild stenosis which does not need correction, and ectopic anus. |
| **Hypospadias (Q54.0-Q54.3, Q54.8-Q54.9)** | A congenital malformation characterized by the opening of the urethra on the ventral side of the penis, distally to the sulcus. Includes penile, scrotal, and perineal hypospadias. Excludes ambiguous genitalia. |
| **Talipes equinovarus/clubfoot (Q66.0, Q66.8)** | A complex deformity of the foot, with three basic characteristics: 1) the affected foot points downward (plantar or equine flexion); 2) the toes point inward (adduction of the foot), and 3) the sole is angled inward (varus deformity of the entire foot). The defect varies in severity and can affect one or both feet. This defect has different names in different countries; it is also known as clubfoot. |
| **Limb reduction deficiencies (Q71.0-Q73.8)** | A congenital malformation characterized by the total or partial absence or severe hypoplasia of skeletal structures of the limbs. Excludes mild hypoplasia with normal shape of skeletal parts, brachydactylic, finger or toe reduction directly associated with syndactyly, general skeletal dysplasia, and sirenomelia. |
| **Exomphalos/Omphalocele (Q79.2)** | A congenital malformation characterized by herniation of abdominal contents through the umbilical insertion and covered by a membrane that may or may not be intact. Excludes gastroschisis, hypoplasia of abdominal muscles, and skin covered umbilical hernia. |
| **Gastroschisis (Q79.3)** | A congenital malformation characterized by visceral herniation usually through a right-side abdominal wall defect to one side of an intact umbilical cord and not covered by a membrane. Excludes hypoplasia of abdominal muscles, skin covered umbilical hernia, and omphalocele. |

^a^International Classification of Disease 10, Royal College of Paediatrics and Child Health (ICD-10 RCPCH) extension for coding of congenital anomalies
